# Artificial Intelligence in the Assessment of Female Reproductive Function Using Ultrasound: A Review

**DOI:** 10.1002/jum.15827

**Published:** 2021-09-15

**Authors:** Zhiyi Chen, Ziyao Wang, Meng Du, Zhenyu Liu

**Affiliations:** ^1^ The First Affiliated Hospital, Medical Imaging Center, Hengyang Medical School University of South China Hengyang China; ^2^ Institute of Medical Imaging University of South China Hengyang China

**Keywords:** artificial intelligence, endometrial receptivity, infertility, ovarian response, ultrasound

## Abstract

The incidence of infertility is continuously increasing nearly all over the world in recent years, and novel methods for accurate assessment are of great need. Artificial Intelligence (AI) has gradually become an effective supplementary method for the assessment of female reproductive function. It has been used in clinical follicular monitoring, optimum timing for transplantation, and prediction of pregnancy outcome. Some literatures summarize the use of AI in this field, but few of them focus on the assessment of female reproductive function by AI‐aided ultrasound. In this review, we mainly discussed the applicability, feasibility, and value of clinical application of AI in ultrasound to monitor follicles, assess endometrial receptivity, and predict the pregnancy outcome of *in vitro* fertilization and embryo transfer (IVF‐ET). The limitations, challenges, and future trends of ultrasound combined with AI in providing efficient and individualized evaluation of female reproductive function had also been mentioned.

**Abbreviations**
AI: artificial intelligence
ANN: artificial neural network
ART: assisted reproductive technology
AUC: area under the curve
ER: endometrial receptivity
IVF‐ET: *in vitro* fertilization and embryo transfer
OR: ovarian reserve
PCOS: polycystic ovary syndrome
RF: random forest
RP: recursive partitioning
SVM: support vector machine
TVUS: transvaginal ultrasound

The incidence rate of infertility is continuously rising worldwide in that approximately one in every six couples suffers from infertility.^1^, ^2^ Assessment of female reproductive function is one of the significant steps in infertility. However, there are many factors which are correlated with the success rate of pregnancy (including age, environment, endocrine level, ovarian reserve, endometrial receptivity, etc.), and the pregnancy results are always caused by various factors.^3^, ^4^, ^5^ The important role of ultrasound in female reproductive function is evaluation of ovarian reserve (OR) and endometrial receptivity (ER).^6^ In the assessment of OR, serial ultrasound examinations can provide reliable markers to follicular monitoring, the diagnosis of Polycystic Ovary Syndrome (PCOS), and prediction of oocyte quality and pregnancy outcomes, such as ovarian follicular diameter and volume, number of follicles, ovarian stromal blood flow index, etc.^7^ For ER, endometrial thickness and volume, endometrial morphology, and spiral arterial blood flow index are effective evaluation indicators.^8^, ^9^ However, the predictive values were still controversial due to the complex markers, limited sample size, and different diagnostic standards among previous studies. In this circumstance, Artificial Intelligence (AI)‐aided diagnosis may be one of the effective solutions.

Over the past few years, AI was regarded as an efficient and reliable method to aid diagnosis, treatment, and prognosis in the medical field, especially after the breakthrough of medical big data analysis and management.^10^, ^11^ Regarding the female reproductive system, a number of studies have focused on the diagnosis and treatment of diseases (such as ovarian cancer and cervical cancer) aided by the AI technique,^12^, ^13^ however, functional assessment is equally important. Currently, AI is currently being tested in several areas of reproductive medicine, including sperm identification and morphology, automatic embryo cell stage prediction, embryo evaluation, and prediction of live birth, as well as the development of improved stimulation protocols.^14^, ^15^ Ultrasound in reproductive medicine, like other disciplines, has been constantly improved by the advances of AI technology. However, there is no consensus on the efficiency and clinical value of AI‐aided ultrasound in the assessment of female reproductive function, and to the best of our knowledge, there is no systematic review which discusses this topic. This paper aims to systematically review the application of AI‐aided ultrasound in female reproductive function. We mainly focused on the application of AI‐aided ultrasound in the assessment of ER and OR, as well as the prediction of pregnancy outcomes (Figure 1). Meanwhile, we briefly discussed its prospects of development. With an increased need in improving the diagnostic abilities and increasing treatment efficiency, it is important to provide efficient and objective acquisition and evaluation of ultrasound images which would bring great benefits to infertile patients.

**Figure 1:**
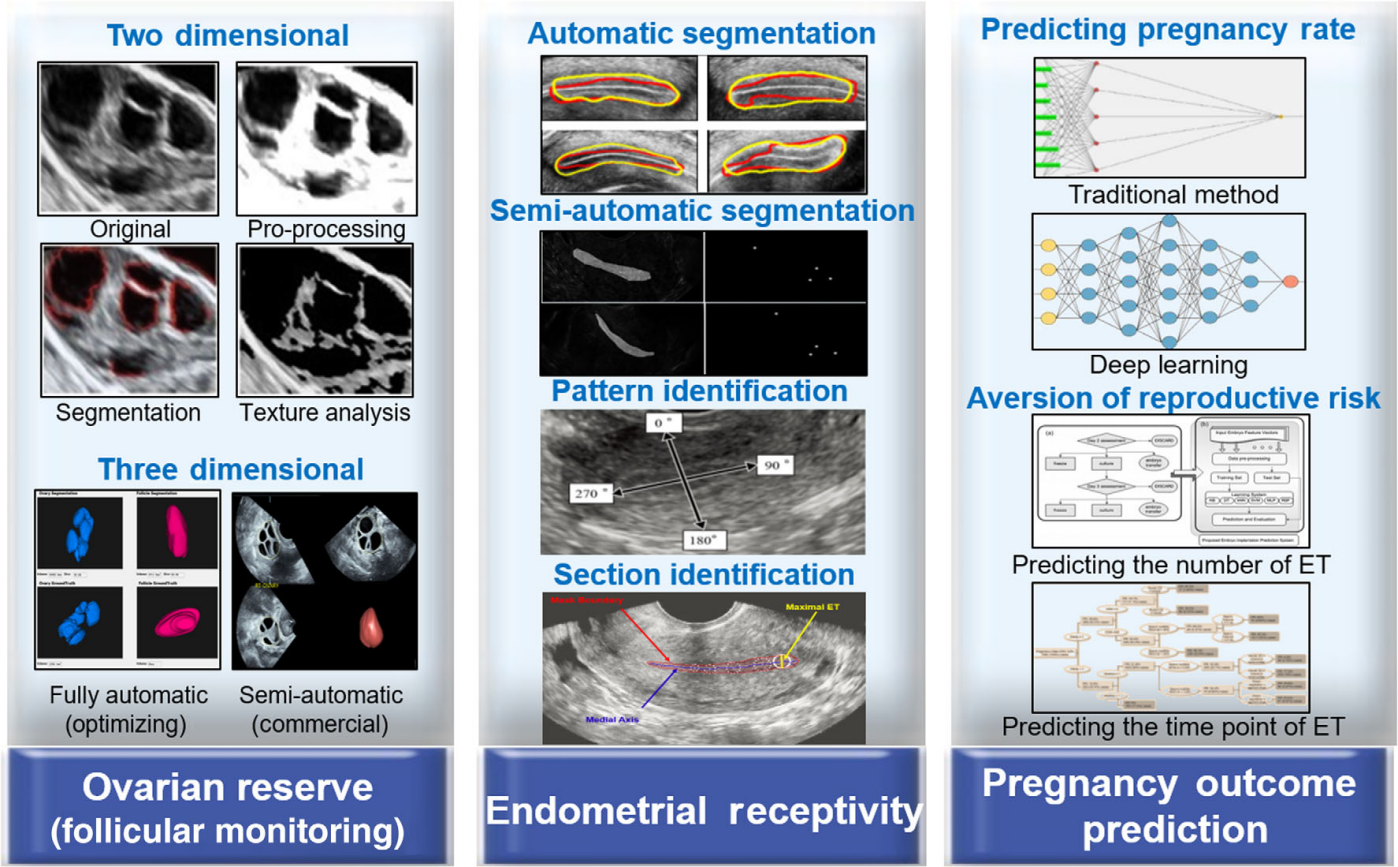
Application of AI in the assessment of female reproductive function. ET, embryo transfer.^45^, ^46^, ^47^, ^49^

## Basics of Artificial Intelligence in Medical Imaging

**Figure 2:**
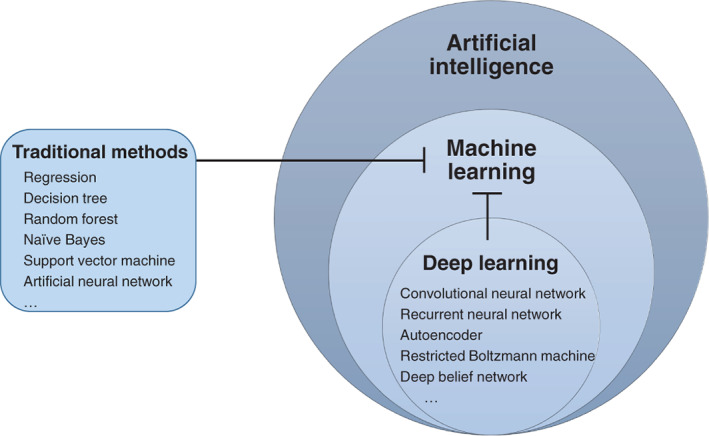
Relationship among artificial intelligence, machine learning and deep learning.

Artificial intelligence in medicine means dealing with the prevention, diagnosis, and cure of diseases through knowledge‐ and/or data‐intensive computer‐based solutions.^16^ Medical imaging is one of the most important application fields for artificial intelligence in medicine, and its first application can date back to the mid‐twentieth century.^17^ We can see artificial intelligence in medical imaging in two perspectives: algorithm method and its clinical application. For algorithm methods, Figure 2 shows the relationship among artificial intelligence, machine learning, and deep learning. Machine learning is a sub‐field of artificial intelligence, which consists primarily of traditional machine learning methods (such as regression, decision tree, random forest, naïve Bayes and support vector machine, etc.) and deep learning algorithms (such as convolutional neural network, recurrent neural network, etc.). According to the aim of study, machine‐learning algorithms fall roughly into supervised and unsupervised. Supervised machine‐learning methods work based on apriori knowledge (a large number of training cases containing inputs and the desired output labels). More specifically, deep learning is a sub‐field of machine learning, which employs artificial neural networks with many layers to identify patterns in huge dataset.^18^ The basic structure of deep neural networks consists of an input layer, a number of hidden layers, and an output layer. ^19^ For the clinical application, AI in medical imaging is commonly used for image segmentation (recognition and segmentation of the region of interest), feature extraction (such as morphological and texture features), and definition of classification systems (disease diagnosis).^20^ Nowadays, for the development of technique and algorithm, AI‐aided ultrasound has become a research hotspot.

## AI‐Aided Ultrasound in Ovarian Reserve: From Accurate Segmentation to Clinical Evaluation

### Application Based on 2D and 3D Ultrasound

**Figure 3:**
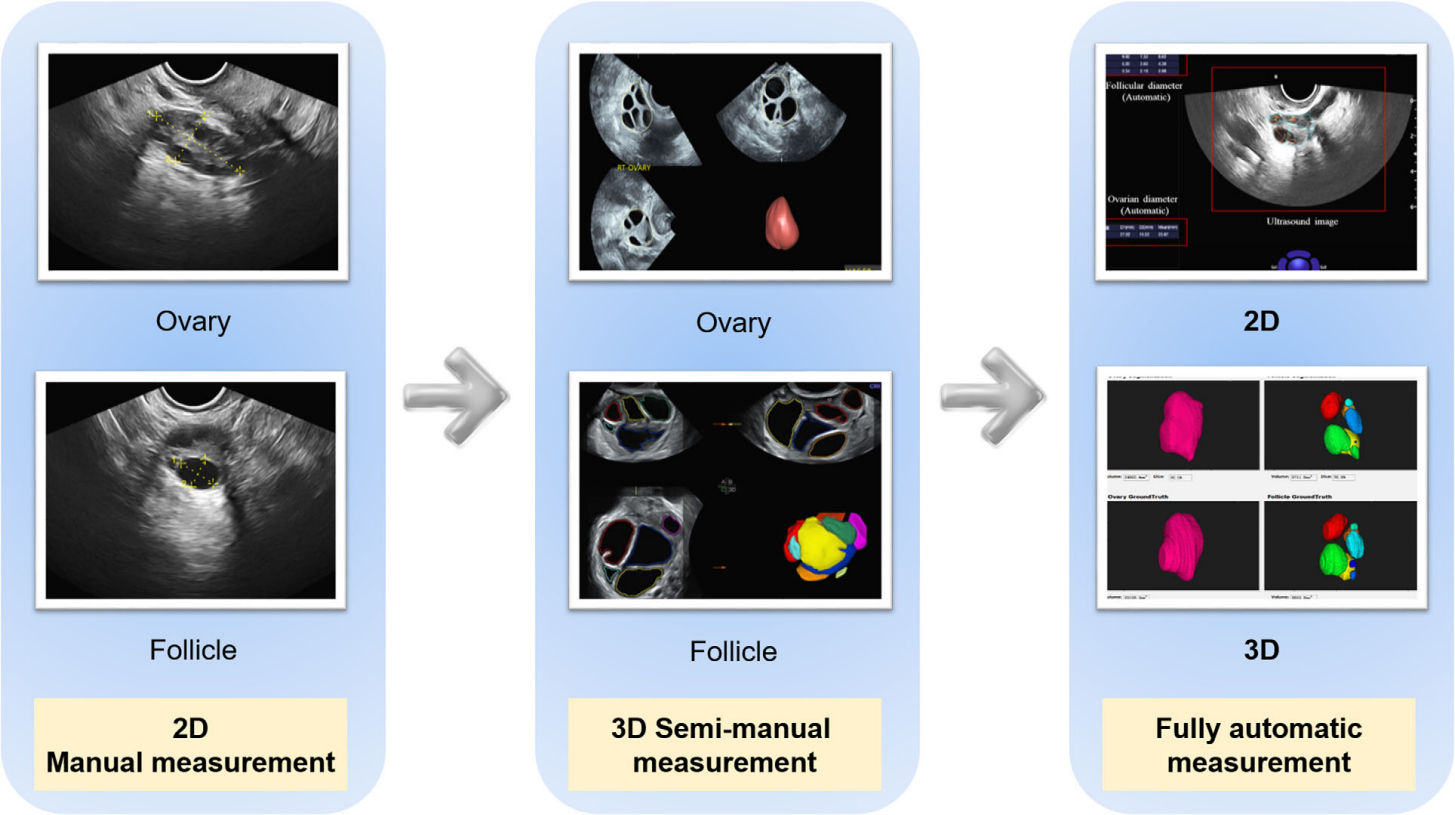
Advanced ultrasound techniques in follicular monitoring.

The first step for the diagnosis and treatment of infertility is to understand the ovarian status and follicle monitoring. Transvaginal ultrasound (TVUS) is an essential diagnostic tool for women undergoing assisted reproductive technology (ART), which can visually observe the development of ovaries and follicles, monitor ovulatory time, and guide the timing of clinical embryo transfer. ^21^ However, it is inconvenient and time‐consuming to perform continuous measurement of follicles and estimation of follicular development during multiple examinations. In the meantime, the inter‐ and intra‐observer difference are significant among clinicians. Since the demand for ART and follicular monitoring is great, AI‐aided ultrasound for the detection of follicles is necessary. As early as 1997, Potocnik et al used an optimal thresholding method to coarsely estimate ovaries and recognize follicles for the first time.^22^ However, the efficiency of this automated method was low (at least six min for processing one image, with a low recognition rate of 70%). Follow‐up studies mainly focused on two aspects; one was optimization of the segmentation algorithms,^23^, ^24^ and another one was the development of new algorithms. Optimal thresholding, edge‐based method, watershed transformation, scanline thresholding, and active contour method were typical algorithms in ovarian follicular boundary segmentation.^25^ Additionally, many studies focused on effectively improving the accuracy of segmentation, shortening the consumed time of segmentation, and verifying the segmentation performance of different algorithms through the same validation set. For example, in view of the limitations of the region growth method which was the need to set the seed point (the origin of “growth” in the algorithm), researchers have adopted an improved discrete wavelet transform‐based k‐means clustering algorithm to improve the accuracy of segmentation.^26^ However, the methods mentioned above were either semi‐automated due to limiting factors, including noise, inability to delineate the boundary of individual follicles, and not being fast enough to be used in real‐time clinical practice. Further strategies on fully automated and rapid segmentation were performed based on image analysis. In Rose's study, follicles were detected by performing different segmentation techniques depending on features of the image (such as pixel intensity level) and features of the areas of detected follicles (such as roundness) to automatic detection of follicles.^27^ In addition, another research conducted texture analysis on ultrasound images of follicles and discovered that the texture features could be used to effectively predict the physiological changes related to future ovulation. This method was conducive to determining the time of oocyte retrieval.^28^ When combined with diagnostic classifiers, these image processing studies could provide novel diagnostic methods for relevant diseases, such as PCOS and premature ovarian failure.^29^ Even so, one issue was that since ovaries and follicles were three‐dimensional organs, two‐dimensional ultrasound imaging could not include all the diagnostic information (Figure 3).

Three‐dimensional ultrasound could be a useful adjunct for follicular monitoring, with a significant reduction in time and a good correlation with manual counts.^30^ The automatic measurements of follicular diameter in 3D ultrasound images seemed to be associated with several advantages when compared to 2D ultrasound images.^31^ Firstly, examination time was reduced because the ultrasound scan data were stored and can be analyzed in detail at a later time. Another advantage was that this new technique reduced the operator's influence on scan interpretation and objectivity; therefore, interobserver variability was reduced.^32^, ^33^ However, it was difficult and time‐consuming for doctors or sonographers to locate the correct planes and measure each individual follicle when there are many follicles in the ovary, even in a 3D ovarian volume. Therefore, an automatic way to detect and measure the follicles is highly desired.^34^ For the improvement of methodology, an algorithm which used multiple concentric layers was proposed in the detection of cattle follicles. The results were compared with the edge‐based method and demonstrated that the proposed algorithm was more effective in follicle detection.^35^ Another research by the same team constructed a system which integrated image denoising algorithm, edge detection algorithm, and 3D reconstruction algorithm. The system showed that monitoring and analysis could improve the success rate of pregnancy outcomes.^36^ In clinical applications, these algorithms were used to estimate the existence of an ovum in the ovarian follicle from ultrasound images^37^ and detect the follicles in stimulation and nonstimulation examination cycles using different ultrasound machines.^38^ It seemed that improved accuracy, faster speeds, and higher robustness was the trend of automatic 3D‐US in follicular monitoring.

### Clinical Implications

No matter if the model was based on 2D or 3D imaging, low generalization was always a big common problem, which limits the clinical application of AI models. In our previous serial studies, we constructed an AI model to segment ovaries and follicles based on the CR‐Unet framework.^39^ The model was experimented on 3204 images with a Dice similarity coefficient of 0.912 and 0.858 in the segmentation of follicles and ovaries, respectively. It was the first work to employ deep learning‐based methods for segmenting both the ovary and follicles in TVUS, and it was proved to have an obvious advantage in the recognition of small follicles (especially <5 mm) over the other state‐of‐the‐arts. Its inter‐ and intra‐observer variability were then validated in a clinical research.^40^ Currently, we are performing this research by loading the software onto the ultrasound equipment. “From laboratory to clinic” for AI‐aided ultrasound is difficult, and the interobserver variability cannot be ignored. To be of high value, the use of the AI‐aided ultrasound model in the assessment of OR should not only focus on the accuracy but also on its clinical utility.

## AI‐Aided Ultrasound: Improving Accuracy in the Assessment of Endometrial Receptivity

In IVF‐ET cycles, inadequate ER is responsible for approximately two‐thirds of implantation failures.^41^ It had been proved that repeated embryo implantation failure, recurrent abortions, and other related diseases were strongly associated with embryo quality and inadequate ER.^42^ Numerous studies showed that various factors such as endometrial thickness and blood supply affected ER, and ultrasound has increasingly been applied for the assessment of ER. However, in the systematic review published in the Human Reproduction Update in 2019, the author used the term “poor ability” to describe the application of ultrasound in the assessment of ER.^43^ The problem may be that sonographers mainly visually observed ultrasound images with their eyes, and manually delineated the boundary of the endometrium for measuring and staging.^44^ Not only was manual segmentation more subjective, time‐consuming, laborious, and poorly reproducible, but also the accuracy of endometrium positioning by junior sonographers was low, and it was easier to make large measurement errors. In recent years, researchers are trying to solve these problems through AI techniques.

AI‐aided ultrasound in the assessment of ER includes segmentation of region of endometrium, classification of endometrial pattern, estimating the accurate motion of endometrium, and assessing the blood supply of endometrium quantitatively. Accurate segmentation of the endometrium is the foundation of a precise measurement. A fully automated segmentation method became the first thought for researchers. The solution provides a performance improvement of approximately 30% over a contemporary supervised learning method on a database of 59 TVUS images.^45^ Since there was no gold standard for identifying the boundary of the endometrium, semi‐automatic segmentation such as setting four key points (endometrium cavity tip, the internal os of the cervix, and the two points between the basal layers on the anterior and posterior uterine walls located on the thickest area) to describe the shape of the endometrium was more accurate than fully automatic segmentation.^46^ Using segmentation based on U‐net, the medial axis transformation method was used to estimate endometrial thickness.^47^ The results were within the clinically acceptable range of 2 mm, which greatly reduced the error of manual measurement. Another ultrasound feature in the evaluation of ER is the endometrial pattern, which can be divided into a trilinear (or leaf), semi‐trilinear, and unilinear (or homogeneous) patterns.^48^ Observation by humans may lead to errors in evaluation, hence AI‐aided ultrasound can be used to automatically identify the type of endometrium. However, subjected to the image quality and inadequate sample size, the efficiency was worse than expected (69.7% for overall accuracy, 60.0% for accuracy of leaf pattern and 78.9% for accuracy of homogeneous pattern).^49^ The result could also be associated with the final outcome, where a number of studies have demonstrated the relationship between the type of endometrial pattern and pregnancy rate.^50^, ^51^

Uterine peristalsis characteristics (or endometrial wavelike activity) has been a research hotspot in recent years. It is caused by the contraction of subendometrial myometrium.^52^ According to the code requirement, sonographers should record a 5 min video for post‐image analysis.^53^ However, it was not convenient for clinical practice. Based on this, Yang et al proposed a new approach for estimating the endometrium based on a multiple threshold technique. His team utilized a recursive algorithm to quickly determine the correct sampling frequency and estimate the accurate motion of endometrium. This method was proven to be successfully and effectively applied to accurately estimate the frequency of motion and the thickness of the endometrium.^54^ For the blood supply of endometrium, Nanni et al developed an artificial intelligence system based on a data mining approach that extracts data from under the endometrium/lumen and evaluates angiogenesis. Age, subendometrial volume, and endometrial vascularization/flow index were observed to obtain the best model for predicting pregnancy rate with an area under the curve (AUC) of 0.85.^55^ In ideal scenarios, automation can reduce the variability in the assessment of ER, thereby enabling consistent and objective measurements to be made. So far, this technique is restricted to the activity of the endometrium, the subjectivity of transvaginal ultrasound, and other factors, and it is just at a research phase. Segmentation, automatic measurement, and diagnosis based on endometrial pattern and automatic recognition of the standard plane are essential steps for this attempt.

## AI‐Aided Ultrasound in Prediction of Pregnancy Outcome: Further Investigation Is Needed

Currently, many infertile couples are under tremendous financial and mental pressure due to the low clinical pregnancy rates and the high cost per IVF‐ET cycle. Predicting the chances of pregnancy in IVF cycles is a long‐standing problem for reproductive scientists.^56^ Therefore, early prediction of the outcomes of pregnancy can guide treatment and reduce the burden of patients. Most of the previous studies combined anti‐Müllerian hormone, antral follicle count, age, and follicle‐stimulating hormone in pairs as effective indicators for predicting pregnancy performance in female reproduction.^57^ However, since pregnancy outcome was affected by various related factors, as well as each factor having individual inaccuracy, the results obtained were different from the actual results and could hardly predict the outcome of pregnancy accurately. Therefore, correlation of the included indicators was closely related to the prediction efficiency of the pregnancy rate, especially for those potential correlations which cannot be easily found.

AI (particularly for deep learning technique) is one of the most effective ways in finding potential correlations.^58^ The first attempt to use the AI technique in predicting the outcome of pregnancy appeared 4 years later than the great assumption of analyzing zona‐free hamster egg sperm penetration by neural network in the animal model.^59^ In that study, indicators of age, number of follicles, number of embryos transferred, and the utilization of embryo freezing were used to construct a neural network model to predict the outcome of pregnancy. The model managed to achieve an overall accuracy of 59%.^60^ Although the accuracy was not satisfactory, and only a few indicators were adopted, the study had laid a foundation for AI‐aided diagnosis in predicting pregnancy outcomes. In terms of how to improve the accuracy, on one hand, the inclusion of more indicators could improve the prediction accuracy in general, no matter what kind of algorithm was used (collecting 27 indicators to build an artificial neural network (ANN) and achieving an accuracy of 90%; or the use of multiple logistic regression analysis to identify risk factors with AUC of 0.78).^61^ On the other hand, the selection of the proper algorithm is equally important.^62^ Hafiz et al verified the accuracy of different classifiers including support vector machine (SVM), recursive partitioning (RP), random forest (RF), adaptive enhancement, and nearest neighbor classifiers on the prediction of pregnancy outcomes in 486 IVF patients. The results showed that the RF and RP methods had good performance of prediction.^63^ However, these results were just the representation of these 486 cases. An interesting series of research demonstrated the whole process of how the AI‐aided method assisted the prediction of the pregnancy rate. The authors proposed the prediction of the pregnancy rate by including majority indicators of IVF cycle assessment (such as stimulation protocol, gonadotrophin dose, hormone level, and couple's information, etc.).^64^, ^65^ However, no further progress has been made due to the difficulties of data collection. In the latest study, this team collected multiple indexes to construct ANN models, where the result showed that ANN models could actually improve the prediction rate of pregnancy outcomes.^66^

Apart from early prediction of pregnancy rate, AI‐aided technique can also help clinicians carry out related reproductive risk aversion. For example, to reduce the risk of multiple pregnancies from the transfer of multiple embryos, Uyar et al expected to provide support for the decision making of the number of embryos to be transferred by predicting the result of single‐embryo transfer in IVF. The study established a traditional Bayesian model with an evaluation accuracy as high as 80.4% and a sensitivity of 63.7%, which was higher than that of expert judgment alone.^67^ Similar conclusions were drawn in a study of fresh periodic single‐embryo transfer (the latter included more cases) published by Blank.^68^ These results were possible for assisting the decision of carrying out or postponing the embryo transfer. Additionally, a study has developed three different approaches (clustering, SVM, and C‐SVM) to predict the cumulative pregnancy rate of IVF in multiple cycles of oocyte pickup using basic patient characteristic, which might help the patient make optimal decisions on whether to use her own oocyte or donor oocyte, how many oocyte pickup cycles she may need, and whether to use frozen embryos, etc.^3^ However, these models which were based on AI techniques have unavoidable shortcomings: unitary application of the model due to small sample size (not applicable to other research samples), incomplete target index (artificial subjective selection), and ineffective pursuit of probability (meaningless probability enhancement). These limitations often lead to distrust of the established models in clinical practice.

To a certain extent, ultrasound‐related information was neglected in previous studies. At present, researchers are exploring the construction of AI models by combining multiple indicators. However, no study has achieved the desired results because the storage and collection of data was extremely difficult, and insufficient sample sizes led to unsatisfactory results. Therefore, collecting sufficient samples and performing data mining analysis on the relationship between the indicators and pregnancy prediction rates are urgent issues in the field of female reproduction. Besides, recognizing that multiple factors, both on the male and female side, may influence the achievement of a successful pregnancy, future deep learning models must not only capture information on embryos, but they must also integrate other relevant patient data.

## Limitations and Strategies

With the advancement of science, AI technology has become increasingly mature and widely used in the medical field. However, current research in assessing female reproductive function is still in the preliminary stage (Table 1). Additionally, there are also ethical issues of responsibility regarding AI technology. Such opacity of lacking a general understanding of the internal processes of AI and human‐machine interaction will produce ethical and liability issues, as well as legal risks, which may lead to the distrust of AI by patients and clinicians.^69^, ^70^, ^71^ Especially in the field of reproduction, most patients do not consent to the uploading of their data to the network for intelligent analysis because of the privacy and security of data.^72^, ^73^ In addition, the performance of AI models was closely related to various factors, including the quantity and quality of the data. If the training data sample size was small, the sample diversity was insufficient, or the ratio among samples was not balanced, this could lead to bias in the model regarding monitoring and learning, which would further result in the low generalization of the models with poor practical application effects.^74^ This problem is more obvious in female reproductive evaluation because of its complicated indicators and uncertainty. Furthermore, high‐quality images and accurate data are the basis of accuracy, which depends on the evaluation standards and the consistent programs of data collecting. A multicenter study is a solution to expand the sample size. Considering the robustness and generalization of the models, data from different machines and different hospitals are needed to construct a highly accurate and high‐performance model.^75^ Since the evaluation standards for female reproductive functions vary, increased efforts should be made to unify the standards and storage methods adopted by different institutions.^76^ Last but not least, the assessment of female reproductive function is very dependent on systematic thinking. This means that despite most of the current research showing that AI can meet or even exceed the performance of experts, it is unnecessary to worry if AI will replace physicians or not. The proper role of AI in the evaluation of female reproductive function should be for screening and early warning. It is important that clinicians should not blindly follow the prediction of the model and should always consider whether the construction of the model is reasonable and whether it is consistent with the actual clinical situation.

**Table 1:** Limitations of AI‐Aided Ultrasound in the Assessment of Female Reproductive Function

Types	Limitation	Results	Solution
Ethical issues	Lack of human‐machine interaction	Lead to the distrust of AI	Informed consent, ensure data safety
	Opacity caused by general understanding of AI's internal processes		
	Data privacy and security		
Problems from images	Lack of quality	Low generalization and diagnostic efficiency	Optimize the instrument, image pro‐processing
	Small sample size		Direct a multicenter study and establish standards
	Ratio among samples is not balanced		
Complexity of reproductive medicine	Lack of universal diagnosis standard	Nonstandard data collection	
	Lack of consistent programs of data collecting		
	Requiring systematical thinking	Cannot reach the clinical problem	Interdisciplinary integration

## Conclusion

In the assessment of female reproductive function, AI‐aided ultrasound is a novel type of interdisciplinary integration.^77^ It will bring digital transformation and automatization to the field of reproductive medicine and will ultimately provide benefits to infertile couples and the society. The combination of AI‐aided ultrasound imaging is conducive to the integration of clinical information, thereby outputting a more objective result, reducing the time of treatment, and providing information for accurate diagnosis and treatment. AI will not replace reproductive medicine practitioners, sonographers, and embryologists, but rather, will streamline their efforts with the goal of better helping their patients. Despite the challenges of application, with the standardized development of technology and the medical industry, AI will assist in the conduct of individualized treatment through the holistic medical information of patients, and its application has unlimited potential for development.
